# Stakeholder Experiences With the Pneumococcal Conjugate Vaccine Chatbot as a Complementary Capacity-Building Tool for Frontline Health Workers in India: Qualitative Study

**DOI:** 10.2196/86326

**Published:** 2026-06-30

**Authors:** Amanjot Kaur, Arindam Ray, Amrita Kumari, Papri Nayak, Jayanta Kumar Sukul, Dimong Padung, Tage Taka, BN Rajani, Syed Sirajuddin Madani, Puneet Jaitley, Amita Raoot, Rhythm Hora, Seema Singh Koshal, Syed F Quadri, Rashmi Mehra, Abida Sultana, Shyam Kimar Singh, Vivek Singh, Arup Deb Roy

**Affiliations:** 1 John Snow India Private Limited New Delhi India; 2 Gates Foundation New Delhi New Delhi India; 3 Ministry of Health, West Bengal West Bengal India; 4 Ministry of health, Arunachal Pradesh Arunachal Pradesh India; 5 Ministry of Health, Karnataka Karnataka India; 6 Ministry of Health, New Delhi New Delhi India

**Keywords:** chatbot, digital health, frontline health workers, immunization, India, pneumococcal conjugate vaccine

## Abstract

**Background:**

Pneumonia remains the leading cause of mortality in individuals aged 5 years or younger globally, with India bearing a disproportionately high burden. The introduction of the pneumococcal conjugate vaccine (PCV) in India necessitated innovative approaches to support frontline health workers (FLHWs), particularly in remote settings. To address this, a customizable WhatsApp-based PCV chatbot was developed as a complementary tool to traditional training and reference materials.

**Objective:**

This study aimed to document the opportunities, challenges, and mitigation measures encountered during the development and rollout of the PCV chatbot, and to explore its use and user experience as a capacity-building and support tool for FLHWs during new vaccine introduction.

**Methods:**

A qualitative study was conducted across 4 Indian states—Arunachal Pradesh, Delhi, Karnataka, and West Bengal—using purposive sampling at the district and block levels. Data collection involved key informant interviews with immunization officials and chatbot developers, and focus group discussions with auxiliary nurse midwives. A Likert scale–based tool captured quantitative feedback on user satisfaction.

**Results:**

Stakeholders appreciated the chatbot’s accessibility, familiarity (through WhatsApp), and multilingual functionality. Most auxiliary nurse midwives found it easy to use and rated responses highly for completeness and usefulness. The chatbot enabled immediate access to information, saving time and bridging gaps, especially when traditional training was delayed or unavailable in hard-to-reach areas. Challenges included occasional technical issues, limited content related to dropout and left-out scenarios, and difficulties in typing regional languages. Recommendations included implementing predictive text, expanding scenario coverage, and strengthening user-centered design and field testing.

**Conclusions:**

The PCV chatbot demonstrated acceptability and perceived value as an on-demand knowledge tool among FLHWs. Continuous user-driven refinement, expanded content, and enhanced usability are essential for its scalability and sustained use in vaccine introduction and capacity-building efforts.

## Introduction

### Overview

Pneumococcal conjugate vaccine (PCV) was introduced under the Universal Immunization Programme (UIP) in a phased manner in India, starting in 2017 from the high burden states and districts [1,2]. The nationwide rollout of the vaccine was completed in October 2021 [3]. PCV scale-up across the country was done during the COVID-19 pandemic, amid the numerous restrictions on gatherings and travel. As is done for all new vaccine introductions, cascade trainings were conducted for the program managers and the frontline health workers/vaccinators. But as the classical classroom model could not be followed for the training, most of these were done in a hybrid or online mode. Therefore, to complement the hybrid mode and to ensure that the vaccinators have the required information about all the aspects of PCV, whenever needed, several initiatives were undertaken, including the development of animated videos and a chatbot dedicated to PCV.

The unprecedented pace of advancements in technology and the increasing amount of time people spend on the internet have led to a major change in lifestyle and community behavior change strategies. As a result, all sectors, governmental and nongovernmental, are constantly evolving and shifting to various digital platforms and solutions to meet user and customer needs. Companies have adopted and implemented information and communication technologies to enhance their relationship channels and make them constantly available in real time to assist customers [4-6].

The PCV chatbot was designed to address queries related to PCV, including dosing, scheduling, administration, cold chain management, storage, side effects, open vial policy, wastage, and eligibility [7]. It was aimed to be very easy-to-use, just like any WhatsApp group, where the auxiliary nurse midwife (ANM) can type in a few keywords related to her query, and the bot will provide a detailed response that includes those keywords. The PCV chatbot was rolled out in select geographies for use by the vaccinators and their supervisors. However, understanding end user perceptions of the PCV chatbot is essential to maximizing its effectiveness and use in the field.

This study aims to document the opportunities, challenges, mitigation strategies, and stakeholder experiences associated with the development and implementation of the PCV chatbot for PCV introduction in India.

### Literature Review

The literature indicates that chatbots are increasingly recognized as effective digital tools to complement capacity building for vaccinators, including in the context of PCV programs [8,9]. Chatbots facilitate on-demand, scalable delivery of vaccine information and guidance, offering interactive support tailored to health care workers’ needs in both routine immunization and outbreak settings [10,11]. Scoping reviews and intervention studies show that well-designed chatbot platforms can enhance vaccine literacy, improve procedural knowledge, and support real-time decision-making for immunization staff, especially in low-resource environments where digital inequities exist [8,9,12].

Notably, chatbot interventions for vaccine confidence and uptake—across COVID-19, human papillomavirus vaccine, and routine immunizations—have demonstrated improvements in both attitudinal and behavioral outcomes, such as increased vaccination intention and actual uptake among end users [10,11,13]. For example, randomized controlled trials in Asia found that chatbot users were more likely to report increased vaccine confidence and acceptance vs nonusers, underscoring their feasibility and acceptability for health communication among frontline workers and the communities they serve [9,10,14].

However, the literature also highlights varied results depending on contextual factors such as chatbot design, conversation quality, and empathy expression by chatbots, which may sometimes negatively affect user evaluation and behavioral intention if not properly aligned with user expectations [15]. Furthermore, process evaluations stress the importance of integrating chatbots with broader digital immunization infrastructure, such as mobile health apps, electronic vaccine tracking, and learning management systems, to achieve sustainable capacity building [12,16,17].

In low- and middle-income countries, deploying chatbots as part of digital training and support strategies has enabled vaccinators to better monitor, analyze, and respond to challenges in vaccine logistics, stock management, and adverse events reporting [16,18]. These innovations empower health care personnel to harness data for targeted interventions, ultimately strengthening immunization systems and coverage [12,19].

Additional references supporting these findings include comprehensive reviews and operational frameworks by global health organizations, which advocate the integration of artificial intelligence and digital platforms to reinforce immunization workforce competencies and real-time vaccine program delivery [17,19,20]. Such evidence emphasizes both the promise and the necessity of leveraging chatbots—and related digital tools—as complementary elements in capacity building for PCV and other vaccine programs.

### Objectives

This study was conducted with the following objectives:

- Objective 1: to document the opportunities, challenges, and mitigation strategies used during the development and deployment of the PCV chatbot.
- Objective 2: to explore the PCV chatbot as a knowledge-building and support tool for new vaccine introduction, capturing end user experiences, perceived benefits, and challenges.

## Methods

### Research Design

**Figure 1:**
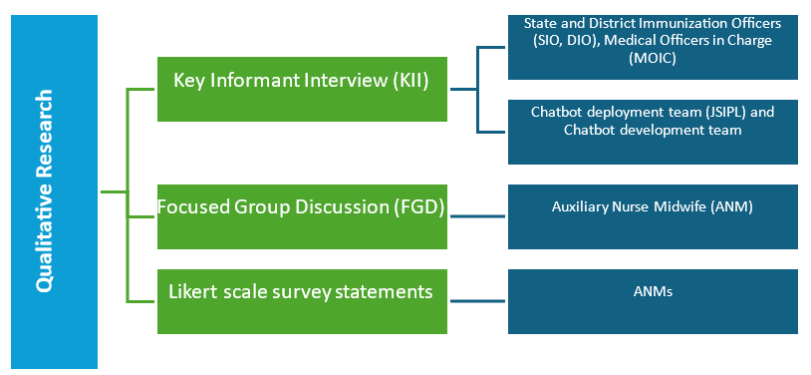
Overall research design. ANM: auxiliary nurse midwife; DIO: district immunization officer; FGD: focused group discussion; JSIPL: John Snow India Private Limited; KII: key informant interview; MOIC: medical officers in charge; SIO: state immunization officer.

A qualitative study design was used, using semistructured interviews to understand the perspectives of key stakeholders regarding the use of a chatbot within the UIP service delivery framework, and specifically for the introduction of new vaccines. Figure 1 presents an overview of the research design. Likert scale survey statements and scoring were also used to gather user feedback on the perceived use and challenges of using the chatbot.

- Key informant interview (KII): the KIIs were conducted with the state, district, and block immunization officials and the PCV chatbot deployment and development team.
- Likert scale survey statements: a short survey was conducted with the ANMs, which included questions based on a Likert scale to understand the satisfaction levels, ease of use, and preference of ANMs to use a chatbot at the time of introduction of a new vaccine. Post this with the same ANMs, the focus group discussion (FGD) tool was executed. This sequence was followed to ensure that ANMs were not biased or collective in their responses.
- FGDs: the FGD tool was conducted with ANMs.

### Study Sites and Sampling

**Figure 2:**
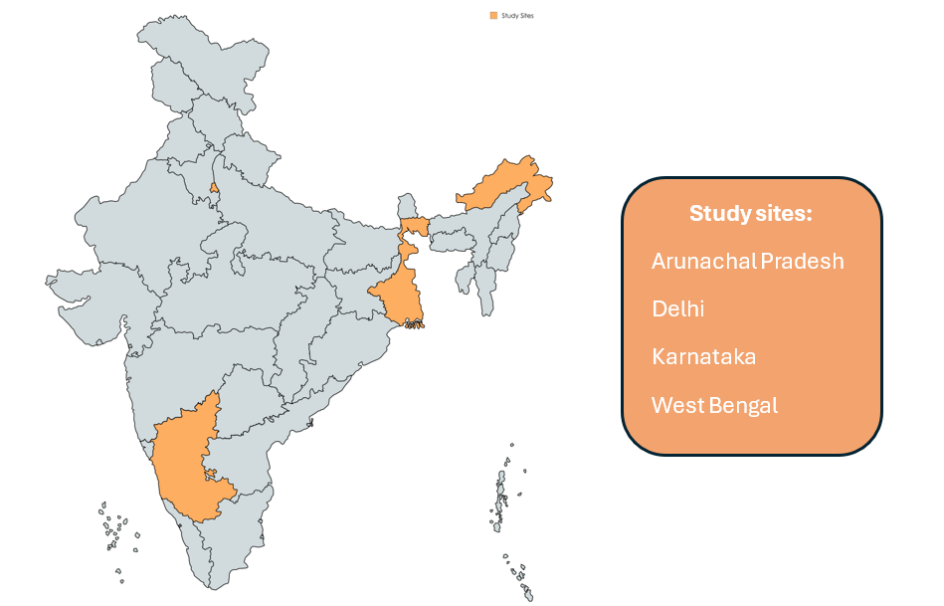
States selected for the research.

The study was conducted in 4 states: Arunachal Pradesh, Delhi, Karnataka, and West Bengal (Figure 2). In each state, two districts were sampled, and within each district 2 blocks were sampled for the purpose of the study. Study states, and sites were purposively sampled based on state official recommendations, John Snow India Private Limited (JSIPL) presence, and logistical and operational feasibility, ensuring geographic diversity and government approval.

**Figure 3:**
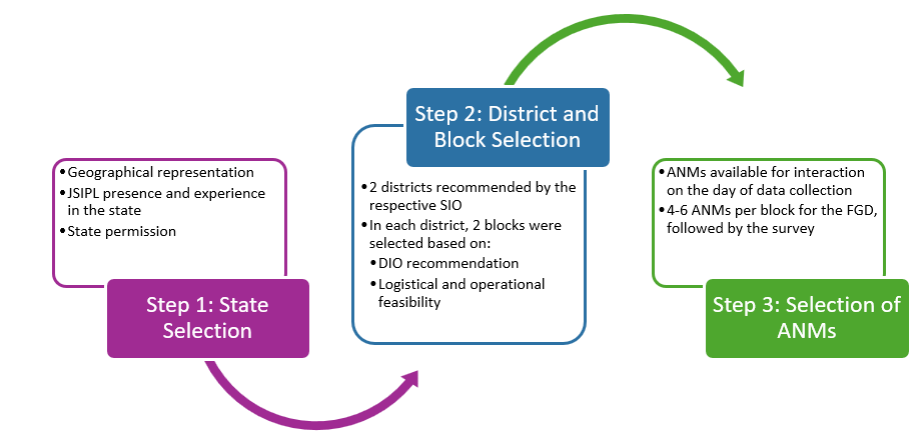
Sampling of study sites and respondents. ANM: auxiliary nurse midwife; DIO: district immunization officer; FGD: focused group discussion; JSIPL: John Snow India Private Limited; SIO: state immunization officer.

The states were selected so as to ensure that the research is not restricted to any one geographical spread in the country. Factors contributing to the state selection also included approvals from the respective state governments to conduct the research in their state, logistical and operational feasibility. For the districts, recommendations from the state officials and the ease of travel to the said district were considered during selection. Two districts in each of the 4 states were selected based on the said criteria. Then, from each district, 2 blocks were selected based on the recommendations of the district officials and operational feasibility. For the FGDs and the short survey, the ANMs who were available for the interaction on the day of the data collection in their respective area were contacted. Thus, this was a purposive sampling for the purpose of the study (Figure 3).

Participant demographics such as age or gender were not recorded, as the study emphasized cadre-specific perspectives (eg, ANMs as frontline vaccinators or state expanded program on immunization officers/district immunization officers/medical officers [MOs] in-charge as immunization in-charges or officials). ANMs were included based on availability at selected facilities, ensuring diverse field experiences across states.

### Sample Size

The respondents included members from the teams that conceptualized the development and deployment of the PCV chatbot, the IT agency that developed the PCV chatbot, and a selection of targeted end users and their supervisors at the block, district, and state levels. The detailed sample size and the theme of the respective enquiry for each category of the respondents are mentioned in Table 1.

**Table 1:** Respondent sample, domains of enquiry, and quantum of data collected.

Level and stakeholder category			Domains of enquiry		Interviews/discussion, n
**National**					
	Program developer of the PCV^a^ chatbot	Process of development of the PCV chatbot, functioning of the chatbot, and addressing postdeployment issues.		1 KII^b^	
	JSIPL^c^ project team	Process of conceptualization, rationale for development of PCV chatbot, inputs and efforts toward developing the PCV chatbot; issues related to the deployment of the PCV chatbot, monitoring, and views on improving the chatbot for future use.		7 KIIs	
**State**					
	SIO^d^	Feasibility of using chatbots by ANMs^e^ during the introduction of new vaccine; views on the effectiveness, usability; possible challenges of chatbot as a knowledge building tool; and recommendation for future use.		4 KIIs (1 per state)	
**District**					
	DIO^f^	Feasibility of using chatbots by ANMs during the introduction of new vaccine; views on the effectiveness, usability; possible challenges of chatbot as a knowledge building tool; and recommendation for future use.		8 KIIs (1 per district)	
**Block**					
	MOICs^g^	Feasibility of using chatbots by ANMs during the introduction of new vaccine; views on the effectiveness, usability; possible challenges of chatbot as a knowledge building tool; and recommendation for future use.		16 (1 per block)	
	ANMs	Feasibility of using chatbots by ANMs during the introduction of new vaccine; views on the effectiveness, usability; possible challenges of chatbot as a knowledge building tool; and recommendation for future use.		16 FGDs^h^ (1 in each block); 74 ANMs participated in the FGDs	

^a^PCV: pneumococcal conjugate vaccine.

^b^KII: key informant interview.

^c^JSIPL: John Snow India Private Limited.

^d^SIO: state immunization officer.

^e^ANM: auxiliary nurse midwife.

^f^DIO: district immunization officer.

^g^MOIC: medical officer in charge.

^h^FGD: focus group discussion.

### Tools

Separate tools were developed for each cadre of the respondents in the study. All the tools developed for the KIIs had sets of open-ended questions covering different themes pertaining to the rationale, design, functionality, content, operability, benefits, and challenges of the PCV chatbot.

In addition to these open-ended tools, a Likert scale–based short quantitative tool was developed for the ANMs, which concentrated on the agreement/satisfaction level of the end users toward the PCV chatbot.

As the ANMs in the field are more comfortable responding and interacting in their local language, the ANM tools were translated into the regional languages for all the 4 states (ie, Hindi for Delhi and Arunachal Pradesh, Bengali for West Bengal, and Kannada for Karnataka).

### Ethical Considerations

As this research involves primary data collection and interactions with human subjects, ethical clearance for carrying out this research was obtained from the Convergent Institutional Review Board (IRB; IRB certificate number Convergent IRB/2024-25/015).

Ethical approval for this study was obtained from the Convergent IRB agency, with certificate number Convergent IRB/2024-25/015 on August 28, 2024.

In addition, a separate permission to conduct the research was obtained from each of the highest state officials.

Consent procedures were in line with those laid out by the ethical certification agency. Study participants were requested to provide informed written and signed consent prior to any data collection. No personal details, such as names, mobile numbers, or addresses of any of the participants, were collected during the research.

It was ensured that any personal identifier information related to the participants was protected, and confidentiality was maintained.

### Data Collection and Management

#### Data Collection

Data collection in all the 4 states was done in September 2024. In order to ensure that there is no data loss due to inability to recall or lack of legible notes, all the interviews were recorded using electronic data recorders (in addition to extensive field notes), after taking written approvals from all the respondents. The data confidentiality of these recordings was also maintained. Personal identifiers were removed from the same before sharing and storing on a secure server, the access to which was limited and credential based.

#### Data Analysis

The qualitative data were analyzed using thematic analysis following Braun and Clarke’s [21] inductive process: familiarization, coding, theme generation via matrices in Microsoft Excel, review, and definition. Themes were derived to represent patterns across stakeholder groups, with representativeness justified by frequency (eg, majority views in FGDs) and triangulation with Likert data. This framework ensures coherent reporting per COREQ (Consolidated Criteria for Reporting Qualitative Research) guidelines. Quotes have been anonymized with role-based codes (eg, ANM-FGD1); selected as representative via frequency in themes (Excel matrices). Full transcripts are stored securely.

## Results

### Overview

Results are structured thematically (1-6), corresponding to study objectives: development opportunities/challenges (Objective 1), and user experiences/benefits (Objective 2).

### Participant Flow and Characteristics

The sample size included 36 KIIs (program managers: n=8; SIOs: n=4; DIOs: n=8; MOICs: n=16) and 16 FGDs (ANMs: n=74).

Demographics were not collected (because emphasis was on role-based sampling), and thus purposive sampling was done by cadre/availability across 4 states.

### Data Analysis and Theme Derivation

#### Overview

The key findings from the thematic analysis are summarized below:

- Inductive thematic analysis (Excel matrices): 6 themes emerged from coding transcripts/field notes, triangulated with Likert data (n=74 ANMs).
- Themes map to objectives: development and deployment of the chatbot (Objective 1) and experiences/benefits/challenges (Objective 2).
- Quotes have been selected as representative of all the participants (majority views per group).

A summary of representative quotes key with corresponding codes, participant cadre, location, and allocated participant number is presented in Table 2.

**Table 2:** Quote key.

Code	Cadre	Description (location)	Participant, n/N
PM^a^-KII^b^ 2	Program manager	National project team	2/8
PM-KII 3	Program manager	National project team	3/8
PM-KII 4	Program manager	National project team	4/8
PM-KII 1	Program manager	National project team	1/8
ANM^c^-FGD^d^ 1	ANM	ANM, FGD in Arunachal Pradesh, District 1 Block 1	4/74
ANM-FGD 4	ANM	ANM, FGD in Arunachal Pradesh, District 2 Block 2	18/74
MOIC^e^-KII-Block 14	MO in-charge	MOIC, KII in West Bengal, District 1 Block 1	14/16
ANM-FGD 10	ANM	ANM, FGD in Karnataka, District 1 Block 2	41/74
SIO^f^-KII	State immunization officer	Since only 1 SIO per state and state names have been mentioned, for anonymity, state name and state code have been redacted	4^g^
ANM-FGD 13	ANM	ANM, FGD in West Bengal, District 1 Block 1	52/74
DIO^h^-KII 3	District immunization officer	DIO, KII in Delhi, District 1	5/8
SIO-KII	State immunization officer	Since only 1 SIO per state and state names have been mentioned, for anonymity, state name and state code have been redacted	4^g^
SIO-KII	State immunization officer	Since only 1 SIO per state and state names have been mentioned, for anonymity, state name and state code have been redacted	4^g^
MOIC-KII-Block 5	MO^i^ in-charge	MOIC, KII in Delhi, District 2 Block 1	6/16
ANM-FGD 7	ANM	ANM, FGD in Delhi, District 2, Block 1	29/74
ANM-FGD 14	ANM	ANM, FGD in West Bengal, District 1 Block 2	57/74
MOIC-KII-Block 3	MO in-charge	MOIC, KII in Delhi, District 1 Block 1	5/16
ANM-FGD 5	ANM	ANM, FGD in Delhi, District 1 Block 1	19/74
ANM-FGD 16	ANM	ANM, FGD in West Bengal, District 1 Block 2	72/74

^a^PM: program manager.

^b^KII: key informant interview.

^c^ANM: auxiliary nurse midwife.

^d^FGD: focus group discussion.

^e^MOIC: medical officer in charge.

^f^SIO: state immunization officer.

^g^Since 4 state immunization officers/state expanded program on immunization officers were interviewed—1 from each state—only the total number is shown to preserve anonymity.

^h^DIO: district immunization officer.

^i^MO: medical officer.

#### Theme 1: Development Rationale

PCV expansion across the country was done in the year 2021, when the world was facing the COVID-19 pandemic, and there were numerous restrictions on gatherings and transportation. Thus, as the lead technical partner supporting the Ministry of Health and Family Welfare in the scale-up of PCV across all states and union territories, JSIPL facilitated the Regional and National level Training of Trainers and supported the states in the cascade trainings as well. Due to the pandemic, this time for the PCV introduction, all these trainings were conducted in virtual or hybrid mode.

As one of the program managers (PM-KII 2) observed and shared:

During field monitoring visits by our state teams, it was observed that the ANMs lacked required knowledge for the introduction of the new vaccine. Since multiple such feedback were received, it encouraged the team to think about exploring the possibility of using technology for the reinforcement of training provided to health care workers.

In order to ensure that the vaccinators had all the required information regarding this new vaccine introduction, a number of innovative tools were developed, which would act as complementary portals for the ANMs/vaccinators where they could access any/all information about PCV. One of these innovations was the PCV chatbot, which was developed as a concept for knowledge building and a support tool for the vaccinators, and was based on frequently asked questions (Textbox 1).

As the turnaround time was very short—PCV was targeted to be scaled-up across the country within a period of 3-4 months—it was only rational to capitalize on the existing technology with which the vaccinators were already comfortable (ie, WhatsApp messenger). It was anticipated that the WhatsApp-based chatbot would not require any additional training or time to teach the vaccinators how to use it.

**Key issues addressed with the pneumococcal conjugate vaccine (PCV) chatbot.**

**Challenges with frequently asked question (FAQ) booklets**

- It was difficult during COVID-19 to ensure that FAQ booklets were provided to the last level of vaccinators.
- Sometimes auxiliary nurse midwives (ANMs) forgot to carry the FAQ booklets to the field, as they had to carry multiple registers, or misplaced them after a few months.

**Reduced time lag in providing PCV-related information**

- In some complex cases, on vaccination days, the ANMs would reach out to their supervisors, but were unable to reach out to them as supervisors were busy with the out-patient department and other patient care services.

**Address gaps in online trainings during COVID-19**

- During COVID-19, online trainings had become the norm. It was difficult for vaccinators to connect and learn from the virtual trainings every time.

#### Theme 2: Development Process

The PCV chatbot was designed with the aim of answering and resolving doubts and issues faced by the vaccinators in the field and was conceptualized to provide a comprehensive knowledge about the newly introduced vaccine (ie, PCV under the UIP). The ministry-approved FAQs for PCV, which are routinely distributed to the vaccinators before the vaccine rollout as hard copies, were used as the basis for developing the chatbot.

A program manager (PM-KII 3), explaining the process of development of the PCV chatbot in brief, said, “We had questions directly from the FAQs translated into the chatbot, which would answer all questions. We also came up with additional questions that we could think of based on our experience.”

Further, ensuring that the program team does not miss out on the latest technological aspects of this tool, the program manager (PM-KII 4) also mentioned that a technical person was included in the team right from the inception of the PCV chatbot, till it went live for use by the vaccinators.

There was an expert in the team who dealt with technology. He was responsible for checking for the content and language appropriateness prior to sending it to the developer.

A detailed master sheet was developed, which had a list of a number of combinations of words, which, when typed in the PCV chatbot, would give a detailed answer related to those keywords. This master sheet was initially developed in the English language. Once it was synced with the PCV chatbot at the backend, to give accurate responses against the respective keywords, the functionality was checked by the team. Once it was ensured that the keywords elicited correct answers, the master sheet was translated into 13 Indian regional languages. This was done for the ease of use by the vaccinators, who are most comfortable in their local language rather than English.

#### Theme 3: Deployment Challenges

The team coordinated with the states for their approval to deploy the PCV chatbot in selected districts in their respective states. A plan was designed to orient the ANMs in the field on the PCV chatbot, its usability, and features, after the approval of the respective state immunization officer. Though it was planned that after an initial pilot in a select district, the PCV chatbot would be rolled out across the states. Even though most states recognized the knowledge gap the chatbot aimed to address, the actual deployment of the PCV chatbot did not receive much traction as governments were prioritizing the response to COVID-19. Additionally, the deployment was marred by technical difficulties, hesitancy among users, and limited usability. The challenges faced during the deployment of the PCV chatbot are captured in Textbox 2.

The challenges in the PCV chatbot deployment in the field were also highlighted by one of the program managers (PM-KII 1):

The other thing was technical glitches that we were facing. When we take something out new in the field, initial glitches are fine, but then it should work smoothly when we give it to the end user. If the end user keeps coming up with glitches and you know, he is not able to get answers. So that kind of compromises our value in the whole immunization programme.

**Challenges faced during the deployment of the chatbot.**

**Technical glitches**

- The deployment faced issues with the server, numbers, and SIM card–related technical glitches that the IT team had to resolve.
- In the field, the pneumococcal conjugate vaccine (PCV) chatbot provided delayed response or no response at all.

**Nonpriority of the government**

- Since the PCV chatbot was launched during the COVID-19 pandemic, the state government prioritized vaccine distribution and controlling the virus’s spread over conducting orientation sessions on the chatbot for auxiliary nurse midwives (ANMs).

**Limited need**

- The PCV chatbot was launched at a time when the ANMs were already familiar with PCV.
- The vaccine was simple to administer, and the ANMs were already familiar with the FAQ based questions of the PCV chatbot.

**Limited usability**

- As the chatbot only addressed PCV-related questions, it limited its utility for the vaccinators.
- The PCV chatbot only provided responses to the FAQ-related questions. It could not respond to the queries related to the issues the vaccinators faced.

#### Theme 4: Stakeholder Experiences

##### Familiarity With Any Chatbot

Out of all the vaccinator respondents, 92% (68/74) had not used any chatbot prior to the study demonstration, and none had heard of the chatbot developed for the PCV introduction. The major reason for this being transfers of the district officials and vaccinators who were present at the time of the vaccine introduction and the deployment of the PCV chatbot. The only respondents who had limited experience of using any chatbots were aware of the ones used for shopping applications.

##### Relevance and Accuracy of Responses

Many respondents did not receive complete or accurate responses to their entered keywords, which created a sense of uncertainty toward the PCV chatbot. According to Figure S2 in Multimedia Appendix 1, 19% (14/74) and 27% (20/74) of ANMs rated the chatbot’s query interpretation as 4 and 5, respectively, on a Likert scale (with 1 being strongly disagree and 5 being strongly agree), indicating concerns about misinterpretation.

Some ANMs, across the states, also felt that the responses to their queries were incomplete, the reason for which was the nonuse of the PCV chatbot for a long time. In the initial phase of PCV launch, the chatbot reportedly provided complete responses to queries, and the response improved with repeated interactions and use by the ANMs.

An ANM (ANM-FGD 1) suggested expanding the chatbot’s keyword database: “The chatbot should include more words in the list of keywords to make it better. For example, the MCP card; there are many suggestions that could be added to the chatbot.”

Despite some instances where the responses were deemed unsatisfactory, the quantitative data suggest that most ANMs (N=74) were satisfied with the completeness of the responses. As shown in Figure S3 in Multimedia Appendix 1, almost 32% (24/74) and 41% (30/74) of ANM gave a high rating of 4 and 5, respectively, for the completeness of the responses provided by the PCV chatbot.

ANMs, when surveyed on their perception of the PCV chatbot’s ability to interpret their query correctly, also displayed a higher degree of confidence. As shown in Figure S4 in Multimedia Appendix 1, almost 20% (15/74) gave a rating of 4, and close to 34% (24/74) rated it at 5. This confidence was also bolstered by the fact that when the exact keywords were entered on different devices, the chatbot was able to respond to the queries in the same manner. They believed that once the PCV chatbot is reworked to respond to “questions asked any which way,” it would interpret their queries correctly.

##### Quality of Content

There were varied responses to the quality of content. When the PCV chatbot responded with the right prompts, MOICs, district, and state-level officials across states were able to understand the content with ease and felt that the answers provided by the PCV chatbot were satisfactory. However, a few MOICs emphasized that the content needed to be less complex and more user-friendly for ANMs. A few other MOs felt that for the supervisors, district and state immunization officers, the content could be updated, and the level of difficulty of the queries could be raised, as their queries would be different from those of the vaccinators.

ANMs, who were sought to be the prime users, were less satisfied with the quality of the content and had several suggestions on the need to simplify the content and provide completeness of responses. ANMs suggested that while the technical language may be correct in the response provided, the content should be easily understandable at the vaccinator level, especially when seeking clarity during the immunization process.

One ANM (ANM-FGD 4) pointed out that “we may need a dictionary to understand everything written here.”

##### Overall Ease of Use

With the demonstration and most getting the chance to use it themselves, stakeholders across the different states felt that the PCV chatbot would be easy to use as it was based on the WhatsApp platform, which they used regularly, both for official and personal purposes. The availability of the PCV chatbot in multiple regional languages was also appreciated by the majority of the vaccinators, as it made the chatbot more accessible and user-friendly. However, a few of the respondents claimed that typing in the regional languages would be different as they were not well-versed in typing using the local language keyboard on their devices.

Another point of view at the level of block MOs was that the PCV chatbot should be flexible, similar to the use of Google, which responds to queries asked using any combination of words.

An MOIC (MOIC-KII-Block 14) suggested that “not only keywords. We should be able to type whatever comes to our mind during that time. Because we cannot keep on searching for the keyword. It comes naturally, like we get whatever we write in Google. So, flexibility should be there. It will be the key for chatbot.”

The state and district level officials felt that the chatbot would be a good supplement to the FAQ booklets, but the inconsistencies and technical bugs need to be addressed before the proper rollout of the tool.

Despite several suggestions, overall, ANMs felt that the PCV chatbot was very easy to use because of its similarity with the basic WhatsApp application.

##### Effectiveness of Use

It is evident in the survey results seen in Figure S6 in Multimedia Appendix 1, where over 48% (35/74) of ANMs suggested that the chatbot would be very useful to facilitate ANMs’ knowledge and performance.

Reinforcing this perception were the survey results for ANM recommending the use of the PCV chatbot to other ANMs, where an overwhelming 66% (49/74) of the ANMs responded that it was “very likely.”

The perception of high effectiveness comes with a few caveats, which have been addressed in the recommendations section of the manuscript.

##### Perceived Use and Benefits

###### Overview

In terms of the benefits and use of chatbots for immunization, several common themes emerged across states. The themes are elaborated below. While most stakeholders were unequivocal about the potential benefit and use, some groups of ANMs, primarily concerned with the possibility of an increase in work burden and extremely poor access to the internet, felt that the chatbot would not be useful. They felt that since they have a good peer support network, they can resolve all their queries related to vaccines and may not need any additional support by way of a chatbot.

An ANM (ANM-FGD 10) compared the PCV chatbot with Google search, saying, “We googled our questions before, but Google does not show just one website. It gives us many options, and we don’t trust the answers.”

###### Accuracy of Information

Since the PCV chatbot was based exclusively on the government-approved FAQ booklets for PCV and given the common use of Google by ANMs for searching for information, SIOs, DIOs, and MOICs across the states felt that having a chatbot that is aligned to the FAQ handbook and regularly updated as per government guidelines would be preferable, as it provides accurate information.

The vaccinators’ confidence in getting an accurate answer was also reflected in the survey result. As shown in Figure S8 in Multimedia Appendix 1, around 45% (33/74) ANMs gave a rating of 5 on how satisfied they felt with the accuracy of the PCV chatbot’s response when they typed in the right queries.

###### Enables Faster Access to Information

Normally, when ANMs have doubts in the field, particularly following left-out and dropout cases of children coming for vaccination, they tend to approach their MOICs over a call to consult on the course of action. However, there are many times when MOICs are busy and unable to take their calls. Under these circumstances, the ANMs must let go of the children as the information comes too late. ANMs shared that when faced with this situation, having a chatbot will be very useful to access information immediately.

Also, as shared by all respondents, although the government provides an FAQ handbook, almost no vaccinator carries it with them to the field, as they must carry registers and other logistics with them to the field. Further, the ANMs do not prefer to use it as it is very time-consuming; for any single query, they must wade through several pages before they get to the answer. Officials at the state and district level also shared that they are very aware of the fact that handbooks are usually left behind at the block level facilities, lying covered in dust. Pointing to the efficacy of a chatbot over a handbook, one of the state officials (SIO-KII) pointed out that, “it’s better to take the soft copy than the hardcopy,” soft copy being the chatbot. ANMs overwhelmingly prefer the chatbot to remain on WhatsApp, citing convenience, storage limitations, and the ability to refer to previous queries.

###### Enhanced Self-Sufficiency of the Vaccinators

As elaborated in the section above, vaccinators mostly rely on their seniors, supervisors, and peers to resolve doubts that arise during administering vaccines in the field. However, as shared by them, if they had an option, they would prefer not to bother anybody else with their queries.

It was found that when the PCV chatbot was demonstrated and explained its objectives, almost 82% (61/74) of ANMs in the survey (Figure S9 in Multimedia Appendix 1) said they would prefer to use a chatbot before reaching out to others for help. This was also reiterated by the MOs, DIOs, and SIOs, who felt that if an ANM had access to a chatbot such as the PCV, it would make them more self-sufficient and improve the quality of services.

An ANM (ANM-FGD 13) quoted, “The PCV Chatbot will be very helpful. Our MOIC tends to be busy. We can immediately ask the chatbot. Sometimes it happens that we want to ask something to our MOIC and if the MOIC does not take the call or responds late, then it becomes a problem for us. We can get the answer from the chatbot immediately.”

A DIO (DIO-KII 3) also put forward the concern of common queries or issues faced by the ANMs in the field. “Common query that ANMs come with are missed doses, delayed vaccination; when to give the next dosage? Such queries can be answered directly by the PCV chatbot if ANM enters the same question. Inclusion of check questions such as: When to start the vaccine, what to do in case of long gaps between vaccines.”

###### Time Saving

Having access to immediate responses using a chatbot was seen to save time both for ANMs, their peers, and their supervisors. Use of the chatbot was also seen to relay timely information to vaccinators when the training was delayed or when outreach to remote areas was difficult. This issue was particularly pronounced in geographically hard-to-reach areas. As pointed out by a few respondents, the training for the rollout of a new vaccine takes time to reach remote areas of the state owing to road connectivity and other issues. Under these circumstances, chatbots that have training material included and videos of the processes of administering the vaccination would be very useful.

Some state officials (SIO-KII) highlighted the network and connectivity issues, which are common in the state, but both are also required for the functioning of the chatbot.

There are connectivity issues in the state because of the geography. Although they do cascade trainings for new vaccines, getting the trainings and information to the vaccinators at all levels gets very delayed. Under these circumstances, a chatbot can be useful. But the ANMs will also need to be trained on how to use the chatbots.

An SIO (SIO-KII) also reemphasized the importance of the internet connection for the smooth functioning of the chatbot, “Chatbot is secondary, but internet connection is a primary need. Internet connection is an issue, especially in the field during outreach activities. This has also affected the U-WIN entries.”

###### An Advocacy Tool for Caregivers

Some ANMs felt that the PCV chatbot should have videos and case studies that could be used to show parents and convince them. It would be particularly useful if parents could be shown what happens to children when they are not vaccinated. Similarly, if they could be shown the adverse events following immunization (AEFI) scenarios, then they would not worry so much if their children got a fever following vaccination. They also shared that beneficiaries having access to information will help build public trust.

An MO (MOIC-KII-Block 5) also echoed this point, saying “ANMS don’t have many questions but parents/caregivers ask lots of questions regarding why, where, when, what is the purpose of giving more than 1-2 vaccines, so sometimes ANMs are unable to resolve all their queries at once. In such cases, they can provide the chatbot number to the caregivers who can then use it for answering their queries.”

An ANM (ANM-FGD 7) also mentioned the point, saying, “Beneficial to provide a chatbot to beneficiaries. Some of the beneficiaries ask a lot of questions and have a lot of queries and if they read and come, a new vaccine would be easier to explain.”

Another ANM (ANM-FGD 14) stated that Accredited Social Health Activist’s (ASHA) work would be easier if the beneficiaries were already knowledgeable about the upcoming vaccines.

Beneficiaries have a right to information too, and if they are provided access, they will be already aware when ASHA go to them and will have less queries. Can show it directly to public—it will build faith among the public that this is a legit vaccine sanctioned by the Government—more effective.

Table 3 summarizes frontline health worker perceptions on familiarity, ease of use, completeness, and likelihood to recommend the PCV chatbot.

The need for a chatbot for use by “beneficiaries,” “clients,” “parents,” and “communities” was reinforced by most officials. The SIOs were keen that the chatbots be available for community use, especially to educate them on AEFI, different scenarios of missed dosage, and the adverse effects of missing vaccination.

**Table 3:** Frontline health worker feedback on the pneumococcal conjugate vaccine chatbot.

Metric	Key finding
Familiarity	92% (68/74) had never used a chatbot prior to the study.
Ease of use	High satisfaction due to the familiar WhatsApp platform.
Completeness	73% (54/74) gave a high rating (4 or 5) for response completeness.
Likelihood to recommend	66% (49/74) were “very likely” to recommend it to peers.

#### Theme 5: Anticipated Challenges

##### Overview

Though there are numerous benefits of the availability and in-use chatbot related to a new vaccine introduction for the vaccinators, like any other intervention, there are some challenges related to the functionality, accountability, and correctness of interpretation of the queries, which were highlighted by the respondents.

##### Availability of Internet Connection and Network

A significant challenge pointed out by the majority of respondents across states and levels was related to internet connectivity and speed. As the outreach immunization sessions are planned and held in the most remote and hard-to-reach areas across states, internet connectivity in such areas is seldom available. Depending on an online application for resolution of queries in such areas might be tricky, and the vaccinators would have to forego caregivers and beneficiaries who come with some special scenario for immunization. That is so because normal phone connections are also very weak in such areas, so the vaccinators cannot call their peers or supervisors as well.

An MOIC (MOIC-KII-Block 3) also said that “when ANMs are in sub-centres, there is internet, so the PCV chatbot can be used there, but might be difficult to use in the VHND sessions.”

##### Access to Smartphones and Digital Literacy

Although a majority of ANMs in all study states reported access to a smartphone and Android phones, access to smartphones and poor digital literacy among older ANMs were considered a challenge. Also, older ANMs and those who live in rural and remote areas may still not be technically savvy enough to use a chatbot. Some ANMs were also reported to have access to a tablet, but not a phone, as they are required to input data in different program applications, which is mostly done in the subcenter. Contextual factors such as a high incidence of phone snatching cases were reported in a few areas where the population is scarce and security is low, which might discourage ANMs from using phones in the field.

An ANM (ANM-FGD 5) also highlighted this point: “pickpockets and phone snatching cases are very common in the vicinity, so ANMs cannot take their phones out and use them in the field.”

##### Possibility of Misinterpretation and Misunderstanding

Long sentences and a lack of uniform interpretation of the keywords and responses provided by the PCV chatbot led to the perceived possibility of misinterpretation of the queries by ANMs. Frequent revision of immunization guidelines by the government may also make it challenging to get the updated information on schedules and protocols, unless the chatbot is updated regularly. ANMs foresee that if the PCV chatbot provides some information that differs from what they explain in nonmedical terms to parents, this could lead to confusion. Remembering correct keywords could be another challenge, as ANMs have multiple workloads and often a short time to use devices in the field; hence, this may lead to entering incorrect keywords and thus result in incorrect responses.

##### Malfunction

Most respondents were first-time users and had little or no previous experience of chatbot use. Hence, breaking of scripts, incomplete and inconsistent messages, repeating the same responses for different queries, insufficient server space, insufficient phone memory to store messages or to operate WhatsApp, and abrupt discontinuation were some of the perceived malfunctions that the respondents anticipated.

#### Theme 6: Disadvantages of Using the Chatbot

##### Overview

While the use of the chatbot offered several advantages, like any emerging intervention, it also presented certain pros and cons. With the pros discussed in the sections above, it is altogether imperative to understand the cons as well, which have been deliberated below.

##### Reduced Human Interaction and Peer Support

Respondents at the state level expressed concerns that reliance on the chatbot could reduce bonding and essential human interaction and engagement between the MOs and DIOs and vaccinators. Additionally, there is a concern that ANMs may become too dependent on the chatbot, potentially disregarding the importance of in-person training for new vaccines. Similarly, a few MOs echoed these concerns, suggesting that the chatbot could inadvertently diminish the importance of adhering to official guidelines and training programs. There is also a perception that officials might become overly reliant on the chatbot, which could lead to negligence in their responsibilities to support field staff. It was also noted that older ANMs, particularly those less comfortable with technology, will not prefer using the chatbot as they continue to find existing systems easier.

##### Create Mistrust Among Caregivers

The use of the chatbot at the session site, in front of the caregivers, has been raised as a significant concern that could lead to potential mistrust in caregivers toward ANMs. The state-level officials and ANMs alike felt that caregivers might question the vaccinators’ capability if they observe the chatbot being used in front of them, potentially interpreting it as a lack of knowledge or confidence in administering the vaccine.

##### Reduced Interest in Training

The state officials across states expressed that while training remains essential, the presence of the chatbot may lead some vaccinators to undervalue structured, in-person training. Concerns were raised that ANMs may start relying too heavily on the chatbot, possibly neglecting the training content they received. Both state and block-level officials suggested that the chatbot should be introduced toward the end of in-person training, and it should be explained to vaccinators that its purpose is to supplement information provided during the training session, and not to be considered as a replacement.

An ANM (ANM-FGD 16) raised a concern of too much dependency on the chatbot, saying, “If everything becomes chatbot related, then slowly the CMHO will lose their importance. Every time they have an issue, and they connect with other ANMs and supervisors—it helps in building a connection. If everything becomes chatbot supported—all these feelings will be lost, we will not need to call them. When we hear their voice, we feel assured and that will be lost.”

##### May Add to the Workload of Vaccinators

Across the 4 states, concerns surfaced that the chatbot might increase the workload of ANMs, particularly among those who are less technologically proficient or who work in areas with unreliable internet connectivity. A few state-level officials highlighted the potential added burden on ANMs if they must juggle the chatbot’s technical requirements alongside their existing responsibilities.

## Discussion

### Key Findings Relative to Objectives

This qualitative study met its 2 objectives through thematic analysis of 36 KIIs and 16 FGDs (74 ANMs) across 4 Indian states. Objective 1 documented PCV chatbot development amid COVID-19 constraints: hybrid trainings revealed ANM knowledge gaps, prompting a WhatsApp-based FAQ tool translated to 13 languages, though deployment hit technical glitches, government priorities, and scope limits (Themes 1-3). Objective 2 captured experiences: 92% (68/74) ANMs were unfamiliar, with high ratings for completeness/accuracy (Figures S2-S4 in Multimedia Appendix 1), ease (Figure S5 in Multimedia Appendix 1), and preference (61/74, 82%; Figure S9 in Multimedia Appendix 1), valuing self-sufficiency over handbooks/Google, but noting internet/digital literacy barriers (Themes 4-6). Overall, chatbots show promise as scalable UIP complements.

### Interpretation, Implications, and Literature Comparison

Globally, as well as in India, there are a number of chatbots being used in the health care system, designed for different end users. Some of these chatbots have been evaluated for their effectiveness, usability, ease of use, reach, and accuracy.

The PCV chatbot was developed and deployed in the pre–generative artificial intelligence period, when such interventions and ideas were not very prevalent. Thus, it was developed so that it was very easy to use, just like any WhatsApp group, where the ANM can type in a few keywords related to her query, and the bot will provide a detailed response that includes those keywords. The evaluation of one such chatbot—the ASHABot—designed to address the information needs of community health workers (CHWs) in India, found that the chatbot provided a private and trustworthy channel for CHWs to ask rudimentary and sensitive questions they hesitated to ask their supervisors. The study suggests that chatbots should be seen as supplemental resources within the community health care system, rather than a replacement for CHWs’ supervisor support [22]. Our study findings highlight a dual perspective on chatbot use among ANMs: on one hand, strong endorsement for enhancing self-sufficiency—82% (61/74) preferred querying the bot before supervisors, citing busy MOICs and instant access (Figure S9 in Multimedia Appendix 1). On the other hand, apprehensions about overreliance, diminishing peer/human interactions essential for nuanced field support, potentially fostering isolation in hierarchical UIP settings. This tension underscores the value of hybrid models that harness chatbots for digital autonomy while preserving essential human interactions and relational training within the UIP framework.

The recommendations collated from the study are summarized below under specific areas.

### Operability

#### Chatbot Should Be Operable and Accessible Offline

Given the issues of internet connectivity faced in all study areas, one of the key recommendations across all states was to enable the chatbot to operate and be accessible offline so that it can be used easily by vaccinators in all types of geographies during immunization.

#### Make Content Accessible to Caregivers

All states reported the hesitancy of caregivers to vaccinate their child. The respondent shared that they have several misconceptions and are misinformed. Making the chatbot accessible to caregivers will help raise their awareness and generate more confidence to get their children vaccinated as per schedule. A study conducted in Argentina aimed to understand if a WhatsApp chatbot, with behaviorally informed functionalities, could improve COVID-19 vaccine uptake [8,23]. The results showed that the chatbot significantly increased COVID-19 vaccine uptake and nearly doubled uptake compared to 1-way message reminders. The findings suggest that communication tools with behaviorally informed functionalities can increase vaccination rates more than traditional message reminders and may have applications in other health behaviors [24,25]. The same has been suggested by some of the ANMs in our study as well; that making a chatbot about the vaccines and their benefits available to the caregivers and beneficiaries would be more beneficial, as it would lead to informed decision-making on their part. Another study supporting the same recommendation shows evidence on the use of a chatbot to support the health-seeking behavior of communities. A study conducted in northern India tested the feasibility and acceptability of “Saheli,” a WhatsApp-deployed chatbot to reach high-risk populations, particularly pregnant and breastfeeding women with targeted COVID-19 information and address low vaccination rates [26]. The study found that a menu-based chatbot was both feasible to implement and acceptable to this population. With high adaptability and scalability potential of chatbots, consideration of deployment strategies and topics to be included was found to be important [27-29]. In another example, with an aim to improve nutrition outcomes for children aged 0-12 months, the Poshan Didi chatbot was designed in Madhya Pradesh through a responsive feedback approach to provide nutrition counseling and information to caregivers. The chatbot created multiple interaction touch points between caregivers (mothers) and health care workers, reinforcing positive behavior change and providing mothers a private platform to discuss topics, which in turn found it receptive, fast, and useful [30].

#### Provide Access to Other Health Workers, Officials, and Beneficiaries With Customized Features

For wider use and acceptance, respondents felt that the application content could be customized to make it usable for MOs, ASHA, and Anganwadi workers as well as beneficiaries.

### Functionality

#### Simplify the Search Function

There were several recommendations made to make the search function for queries simple and easy to access precise content. These include:

- Enable predictive text: rather than having to remember and raise the query using keywords only, respondents across the spectrum recommended that the chatbot should be able to offer predictive text when the first few words of the query are typed in, such as in Google search or WhatsApp text. It should be able to understand partial questions or long sentences and provide accurate answers.
- Provide a content page from which the required topic can be easily accessed: some respondents suggested that, since the content of the chatbot is predetermined, the search function should be enabled using a content page where all topics can be read at a glance, and the required topic can be accessed through a “click,” linking it to the content page.
- Enable the chatbot to read queries as articulated by the user, using any combination of words. Given that the chatbot will be designed for use by vaccinators with limited time and language competency, the chatbot should accept queries as articulated by them and not only through keywords.

#### Enable Live Chat Services

The chatbot currently has fixed content and is aligned to the FAQs that most vaccinators and their peers are familiar with. However, vaccinators during immunization are faced with complications and problems that may be beyond the ambit of the FAQs. Thus, it was recommended by ANMs in Arunachal Pradesh that a live chat service should be enabled so that vaccinators can get responses for queries not available in the chatbot. As an ANM clarified, “like customer service, a person should be there on the other end who can respond to our queries in real time.”

#### Use Simple and Easy-to-Read Language

Respondents suggested that the current language of the content is quite technical and may not be easy to understand at first read.

#### Make the Chatbot Voice-Enabled

Respondents across the spectrum recommended that the chatbot be voice-enabled, where the vaccinator can ask a question through a voice note and get the answer in a similar way. This would also enable older ANMs with limited digital literacy to operate it easily.

#### Develop an Offline Chatbot

For areas with limited or no access to the internet, the chatbot requires facilitating conditions such as internet connectivity, reliable and steady internet in the field, and technical support to be effective. Without these, users may be less likely to adopt or continue using the chatbot. Conversely, if users have access to necessary resources and support, they may be more inclined to integrate the chatbot into their daily immunization routines and continue using it. Therefore, ensuring these conditions are met is crucial for a chatbot’s success. In the Indian context, the chatbot should be designed to function both online and offline, with live chat features to address unforeseen issues related to vaccination scenarios.

### Content

While all respondents were largely satisfied with the content, there were several recommendations on enhancing the existing content and adding new content.

#### Include Audio-Visual Content

It was recommended at all levels that the chatbot add audio-visual material, especially related to the process of administering a vaccine and managing AEFI.

#### Include Information for All Vaccines Included in UIP

Officials and vaccinators across states recommended that if the chatbot provided information for all vaccines, the use and utility of the chatbot would be highly enhanced. As this chatbot was developed specially for the PCV vaccine, respondents felt that it would be much more useful if the entire module on all vaccines under the UIP could be included within the chatbot. This would not only serve as a refresher for health workers working in the sector but also serve as training material for new ANMs. ANMs felt that introducing all vaccine schedules also helped in linking information related to existing vaccines with new vaccines. Having an entire gamut of immunization-related information would also provide a holistic picture to the users, provided that the information was regularly updated.

#### Include Content Beyond What Is Provided in the FAQs

SIOs and DIOs recommended that a chatbot should not only be restricted to FAQ related questions but also be able to respond to real-time issues and complications faced by vaccinators during immunization. Some of the recommendations included the following:

- Develop a chatbot-based pan-India survey on the frequent queries of vaccinators. An SIO suggested that to ensure that chatbots can respond to real-time issues for vaccinators, it may be useful to conduct a pan-India survey among vaccinators to understand the key issues faced during immunization and develop the chatbot to provide those responses.
- Include all possible scenarios for dropped out and left out cases: an SIO suggested that the chatbot should include responses for all scenarios of cases of children who drop out or miss the immunization schedule, and thus may be beyond the prescribed age for the required dose. He suggested that these scenarios can be drawn from the FAQs. All MOICs and vaccinators also reiterated this need as the maximum queries from the field pertain to this issue.

#### Include Content That Can Be Used for Advocacy With Caregivers

Respondents at all levels, across the 4 states, recommended that chatbots should include content that vaccinators can use to show and inform caregivers, particularly those who are hesitant to vaccinate their children or are misinformed about AEFIs. ANMs across states reiterated the need for this content as one of the key issues faced by them is the lack of understanding among parents on the importance of vaccination and the significance of sticking to the schedule. They felt that if they used the chatbot with content directly responding to caregiver fears and misconceptions, then it would inspire more confidence in them.

#### Improve the Completeness of Responses

ANMs across states recommended that if they are to rely on the use of chatbots officially, then the responses should be accurate and more complete than what they experienced during the demonstration. For example, when an ANM asked about the cost of the PCV vaccine, the chatbot responded that it was expensive but did not provide the actual cost.

#### Avoid a “Sorry” Response

It was recommended that under no circumstances should the chatbot offer a “sorry” answer. As recommended by a DIO, the chatbot should be able to respond to questions asked in any manner. It should not give a sorry for an answer. If an answer is not known, then a related link should also be available.

### Monitoring and Accountability

In all states, following the discussions on monitoring and accountability, outlined below are the recommendations made by different levels of respondents.

Update regularly as per the government guidelines: all respondents recommended that it would be mandatory for the chatbots to be updated as per the new guidelines and protocols that are introduced by the government for the same vaccine. If this is not done, the chatbot will become more of a liability than an aid.

### Limitations of the Study

This study has several limitations that should be considered when interpreting the findings. First, the purposive sampling strategy, particularly for ANMs based on availability at health facilities during data collection, constitutes convenience sampling, which limits generalizability beyond the selected sites in Arunachal Pradesh, Delhi, Karnataka, and West Bengal. While states were chosen for geographic diversity and logistical feasibility, findings may not fully represent all UIP contexts, such as high-burden states or union territories.

Second, no demographic data (eg, age, gender, and years of experience) were collected from participants, as the focus was on cadre-specific perspectives. This assumes relative uniformity within roles (eg, state expanded program on immunization officers/district immunization officers/MOs in-charge as MBBS physicians, ANMs as frontline vaccinators), which may overlook variations in digital literacy or tenure affecting chatbot perceptions, particularly among older ANMs noted in qualitative responses.

Third, data collection occurred in September 2024, over 3 years post–PCV rollout (2021), relying on retrospective recall. This introduced potential recall bias, evidenced by 92% (68/74) ANM unfamiliarity with the chatbot, largely due to staff transfers common in public health systems. Real-time experiences during deployment may have differed, though field monitoring notes from program managers provided contemporaneous corroboration.

Fourth, the mixed methods sequence—Likert surveys preceding FGDs with the same ANMs—may have introduced response bias, as quantitative priming could influence qualitative discussions toward positive framing. While this order minimized groupthink in FGDs, reverse sequencing (FGDs first) might have yielded more unprimed views.

Fifth, the study focused exclusively on the PCV chatbot, limiting insights into multivaccine or routine immunization applications. Stakeholder suggestions for broader UIP integration remain untested empirically.

Additional methodological constraints include the hybrid nature of PCV trainings influencing baseline knowledge gaps, and technical demonstrations during FGDs potentially inflating perceived ease-of-use ratings (Figures S5-S9 in Multimedia Appendix 1). Finally, as an implementation study by chatbot developers, researchers’ familiarity with stakeholders risked social desirability bias, though ethical protocols (eg, anonymity and external IRB) and triangulation mitigated this.

Despite these limitations, the large sample (n=110), multistate design, and convergence of qualitative themes with Likert data enhance credibility and transferability to similar low- and middle-income country vaccine introductions. Future prospective, multivaccine trials could address these gaps.

### Conclusions

This study affirms WhatsApp-based chatbots like PCV chatbot as viable, low-cost complements to cascade trainings for new vaccine introductions in India’s UIP, particularly during disruptions such as COVID-19, by enabling on-demand, multilingual access that 82% (61/74) of ANMs preferred over supervisor calls for routine doubts. Beyond reinforcing knowledge gaps and self-sufficiency, findings reveal untapped potential for community advocacy, positioning chatbots as equity tools for remote caregivers hesitant about AEFI or schedules.

Broader implications extend to UIP resilience: integrating predictive, voice-enabled, offline-capable bots for multivaccines could standardize field support nationwide, reducing dropout rates in hard-to-reach areas where connectivity lags. National policies should prioritize user-centric design—audio-visual content, real-time updates—drawing from successes like ASHABot, potentially lifting coverage by 10%-20% per Asian trials while preserving peer networks.

For low- and middle-income countries, this model offers a scalable blueprint: government-approved FAQs via familiar platforms bypass digital divides better than apps, fostering ANM autonomy without overreliance. Future innovations—artificial intelligence analytics on queries, caregiver portals—could transform immunization from reactive to predictive systems, advancing Sustainable Development Goals 3 targets amid climate/geopolitical shocks.

## Appendix

Multimedia Appendix 1 Supplementary material.

## Abbreviations

AEFI: adverse events following immunization
ANM: auxiliary nurse midwife
ASHA: Accredited Social Health Activist
CHW: community health worker
COREQ: Consolidated Criteria for Reporting Qualitative Research
FGD: focus group discussion
IRB: institutional review board
JSIPL: John Snow India Private Limited
KII: key informant interview
MO: medical officer
PCV: pneumococcal conjugate vaccine
UIP: Universal Immunization Programme
