# Supplementary material for: Stakeholder Experiences With the Pneumococcal Conjugate Vaccine Chatbot as a Complementary Capacity-Building Tool for Frontline Health Workers in India: Qualitative Study
Source: JMIR Form Res. 2026 Jun 30;10:e86326. doi: 10.2196/86326 (PMC13370108; doi:10.2196/86326)
Supplement: Multimedia Appendix 1 [file formative_v10i1e86326_app1.docx]

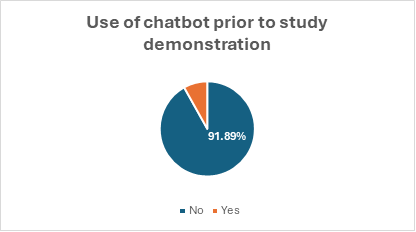


*Figure S1: Familiarity with any chatbot*

**N = 74**

*Figure S2: Misinterpretation of questions by PCV chatbot*


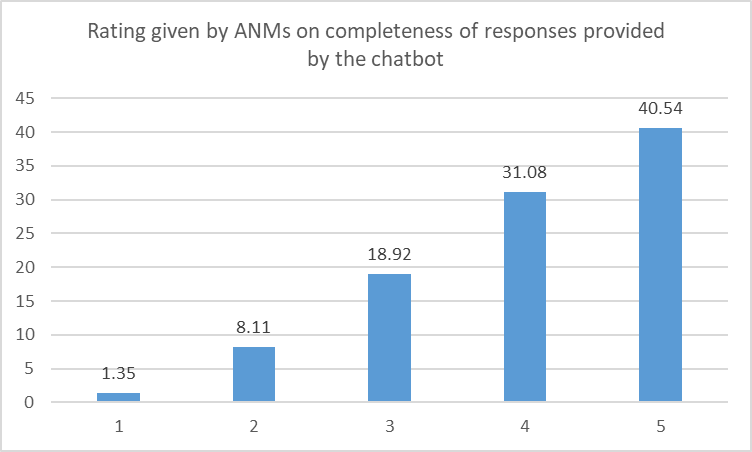


*Figure S3: Completeness of responses*

**N = 74**


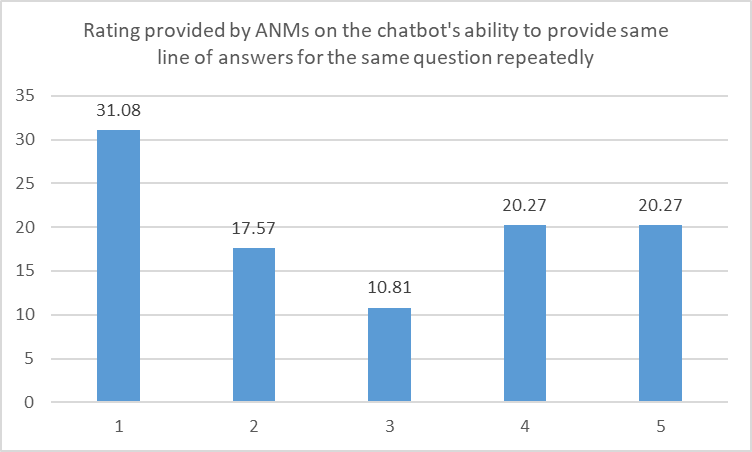


*Figure S4: Accuracy of Responses*

**N = 74**


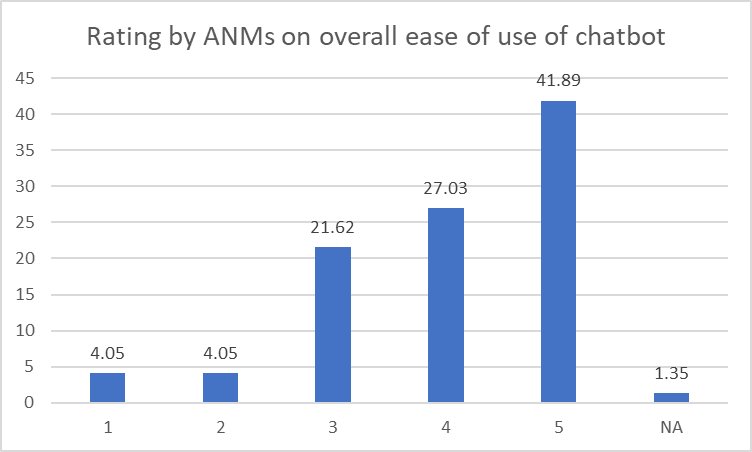


*Figure S5: Ease of use*

**N = 74**


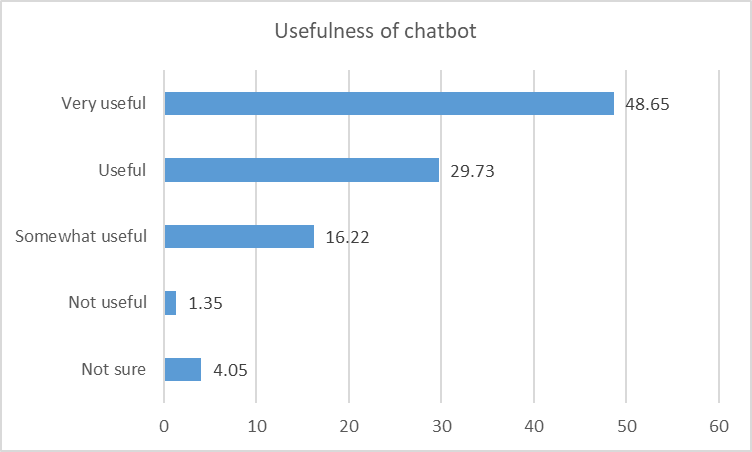


*Figure S6: Usefulness of a Chatbot (percentage of ANMs)*

**N = 74**


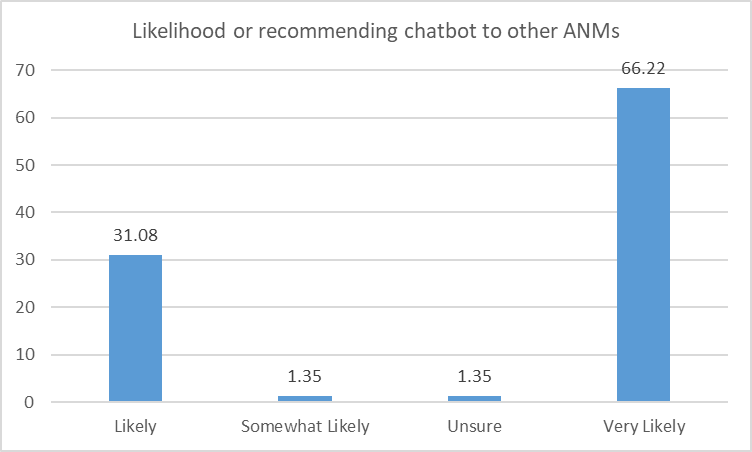


*Figure S7: Likelihood of recommending PCV chatbot to other ANMs*

**N = 74**


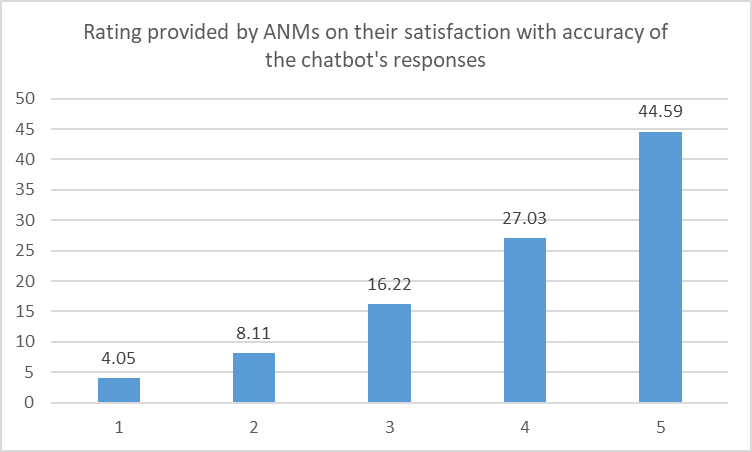


*Figure S8: Accuracy of the PCV chatbot*

**N = 74**


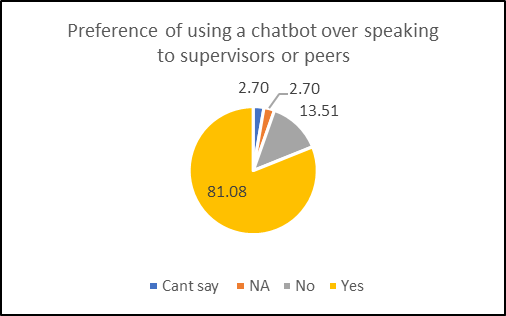


Figure S9: Preference of using chatbot by ANMs
